# Adénocarcinome vaginal primitif de type intestinal: cas clinique et revue de la littérature

**DOI:** 10.11604/pamj.2013.15.30.2301

**Published:** 2013-05-21

**Authors:** Said Ait Laalim, Imane Tourghai, Karim Ibnmejdoub, Khalid Mazaz, Sanaa Raghy, Leila Chbani, El Mazghi Abderahman, Fatim Zahra Hijri

**Affiliations:** 1 Département de chirurgie générale, CHU Hassan II, Fès, Maroc; 2 Département de gynécologie obstétrique, CHU Hassan II, Fès, Maroc; 3 Département d'anatomo-pathologie, CHU Hassan II, Fès, Maroc; 4 Département d'oncologie, CHU Hassan II, Fès, Maroc

**Keywords:** Adénocarcinome vaginal, type intestinal, radio-chimiothérapie, chirurgie, mauvais pronostic, vaginal adenocarcinoma, intestinal type, chemoradiotherapy, surgery, prognosis

## Abstract

L'adénocarcinome vaginal de type intestinal est une tumeur rare. Il pose un problème d’étiopathogénie, de diagnostic différentiel (avec un ADK rectal ou métastatique) et de prise en charge thérapeutique. Nous rapportons le cas d'une patiente de 46 ans, qui présente depuis 8mois des leucorrhées brunâtres. L'examen clinique trouve la présence d'une lésion infiltrante de 4cm, au dépend de la paroi postérieure du vagin avec envahissement de la cloison recto-vaginale. La biopsie a révélé un adénocarcinome colloïde muqueux de type intestinal. Cette interprétation histologique orientait vers une métastase vaginale d'un ADK gastro-intestinal, cependant, l'examen clinique, la recto-coloscopie, le scanner abdominal et l'imagerie par résonance magnétique (IRM) pelvienne ont permis d'exclure cette possibilité. La patiente a reçu une radio-chimiothérapie concomitante (RCC), suivie d'une résection chirurgicale. L'examen anatomopathologique de la pièce opératoire a montré une stérilisation totale de la tumeur. Après 2 ans de suivi la patiente a développée des métastases osseuses multiples témoignant de l'agressivité de cette tumeur.

## Introduction

Le cancer primitif du vagin est une tumeur rare, il représente 1à 3% des cancers gynécologiques et touche surtout la femme âgée [1]. Histologiquement, 95% des tumeurs primitives malignes du vagin sont de type épidermoïde [2]. Les adénocarcinomes (ADK) de type intestinal sont exceptionnels et seulement 9 cas ont été rapportés dans la littérature. Ce type de cancer pose un problème d’étiopathogénie, de diagnostic différentiel (avec d'autres ADK rectal ou métastatique), et de prise en charge thérapeutique. Nous relevons le défi diagnostic et thérapeutique de l'ADK primitif du vagin de type intestinal à travers un dixième cas et une revue de la littérature.

## Patient et observation

**Figure 1:**
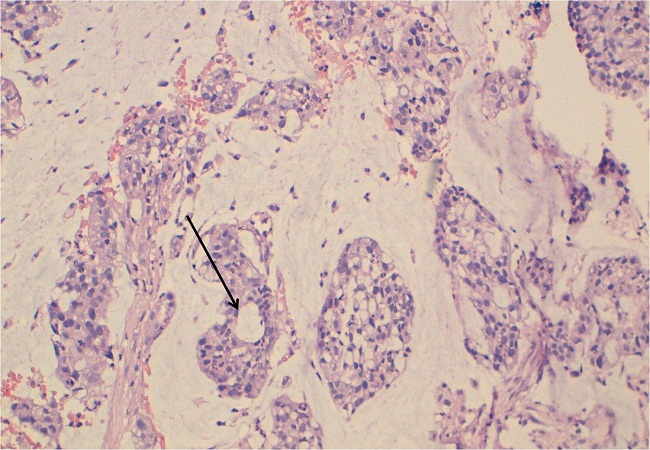
HESx250: prolifération tumorale adénocarcinomateuse (flèche) au sein de plages de mucus

Patiente de 46 ans, célibataire, sans antécédents d'endométriose ou d'exposition maternelle au Diethystilbestrol (DES). Hystérectomisée il y a 2 ans pour utérus polymyomateux, sans signes de malignités à l'examen anatomopathologique de la pièce opératoire. La patiente a présenté depuis 8mois une douleur périnéale à type de pesanteur associée à des leucorrhées brunâtres. L'examen clinique a révélé la présence d'une masse infiltrante, dure, blanchâtre et mal limitée au dépend de la jonction tiers inferieur/tiers moyen de la paroi postéro-latérale gauche du vagin. Le toucher rectal a montré la présence à 4 cm de la marge anale d'un bombement de la paroi antérieure du rectum évoquant une compression extrinsèque par une tumeur de voisinage. L'examen des aires ganglionnaires a révélé l'existence d'adénopathies inguinales bilatérales de plus de 1 cm pour la plus grande. Un examen sous anesthésie générale (pour mieux apprécier l'extension locale de la lésion) associé à une rectoscopie a montré que la tumeur a été au dépend de la paroi postérieure du vagin, envahissant la cloison recto-vaginale (CRV) sans atteindre la muqueuse rectale. Cette dernière a été d'aspect normal et sans signes de malignités sur les fragments biopsiés. Par contre la biopsie vaginale est revenue en faveur d'un ADK colloïde muqueux, franchement marqué par l'anti-corps anti-CK 20 et négatif au CK7 et la CK5/6 concluant ainsi en un ADK mucineux d'origine digestive (Figure 1). A l’écho-endorectale la lésion tumorale a été au dépend de la CRV sans atteindre la muqueuse rectale, d'autres biopsies ont été réalisées sur le versant rectal, qui sont revenus négatives. L'imagerie par résonance magnétique (IRM) pelvienne montre qu'il s'agit d'une lésion de la paroi postéro-inférieure du vagin envahissant la CRV, s’étendant sur une longueur de 5cm et mesurant 18mm d’épaisseur par endroit.

**Figure 2:**
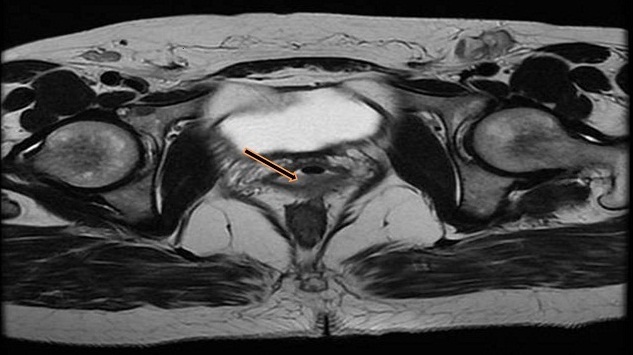
IRM pelvienne montrant une lésion de la paroi postéro-latérale gauche du vagin (flèche) envahissant la cloison recto-vaginale

**Figure 3:**
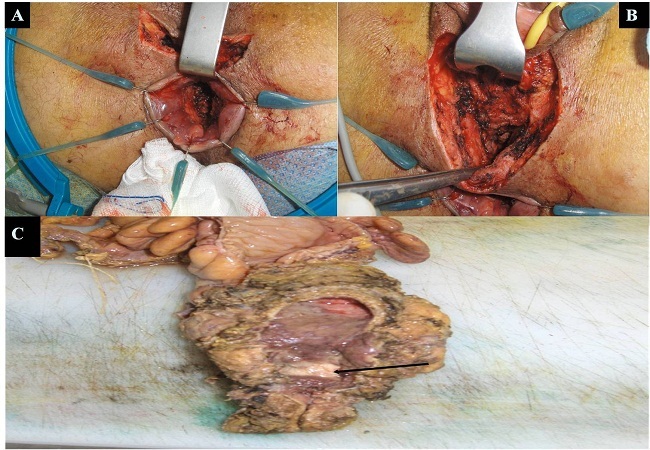
A) Image per opératoire montrant la résection intersphinctérienne par voie périnéale. B) Image per-opératoire montrant la résection de la paroi postérieure du vagin. C) Photo de la pièce opératoire, montrant une lésion blanchâtre de la paroi postérieure du vagin (flèche), qui correspond à l'adénocarcinome vaginale après radio chimiothérapie néo adjuvante

Cette lésion s’étend en arrière à l'espace ano-vaginale et envahit par endroit le canal anal du côté gauche à 2.5cm de la marge anale (Figure 2), avec présence d'ADP inguinales bilatérales. La tumeur a été classé stade III selon la classification FIGO. Après avoir éliminé un ADK d'origine rectale, notre attention a été orientée au début vers une métastase vaginale d'un ADK digestif. Un bilan a été réalisé dans ce sens (coloscopie totale, scanner abdomino-pelvien) et qui a été sans particularité. Les marqueurs tumoraux (ACE, CA19-9) étaient normaux. Le diagnostic d'un ADK vaginal primitif de type intestinal a été retenu et la patiente a reçu une radio-chimiothérapie concomitante (RCC): radiothérapie pelvienne à la dose totale de 45Gy en 25 séances de 1.8 Gy, étalée sur 5 semaines ainsi qu'une radiothérapie inguinales bilatérales. Cette radiothérapie était associée à une chimiothérapie à base de capécitabine (Xeloda) à la dose 825mg/m^2^ les jours de la radiothérapie, puis elle a été opérée à la 6éme semaine post RCC. Il a été réalisé une résection inter-sphinctérienne (RIS) et de la paroi postérieure du vagin avec un curage inguinal bilatéral (Figure 3). Les suites opératoires ont été marquées par l'installation d'une fistule digestive périnéale, des fuites urinaires insensibles et des rétentions d'urines nécessitant un recours à l'autosondage. L'examen anatomopathologique de la pièce opératoire a montré la présence d'une induration de 4/4cm au dépend du 1/3 moyen de la paroi postérieure du vagin. La réponse thérapeutique a été sous forme de flaque de colloïde sans résidu tumoral. Les Limites chirurgicales sont non envahies. Pour le curage ganglionnaire, il y avait 18N- /18N sur la pièce opératoire et 8N-/8N sur le curage inguinal.

La tumeur a été classée ypT0N0M0. La patiente a reçu en adjuvant 6 cures de Folfox 4 simplifié (oxaliplatine + LV5FU2 simplifié), et devant la persistance de la fistule digestive et la mauvaise cicatrisation périnéale, une graciloplastie a été réalisée comblant cette perte de substance et protégeant la suture colique. Après 2 ans de suivi la patiente a développée des métastases osseuses multiples, rachidiennes et costales.

## Discussion

Le cancer primitif du vagin est une tumeur rare et représente 1 à 3% de tous les cancers gynécologiques [1]. L'ADK est une entité anatomopathologique rare et se voie dans 14% des tumeurs primitives du vagin [3]. Parmi ces tumeurs, les ADK à cellules claires sont les plus fréquemment décrits, ils surviennent chez la fille jeune et sont généralement associés à un traitement de la mère au cours de la grossesse par le DES [1]. Les ADK de type intestinal sont exceptionnels et seulement 9 cas ont été publiés dans la littérature [4–11] (Tableau 1).

**Table 1:** Adenocarcinome de type intestinal primitif du vagin. Revue des 9 cas rapportés dans la littérature

Nom	Année	Age	Symptôme	Localisation	Taille cm	Origine	FIGO	Traitement	Suivie
**Fox et al (4)**	1988	35	PV	PAV	5 x 2,4 x 3,5	Cloacal	I	PA	6 mois/Pas de récidive tumorale
**Yaghsezian (5)**	1992	52	SV	PPV	1	AV	I	LE +RT	8 mois/vivant avec la maladie
**Nagar et al (6)**	1999	36	SV	PAV	NA	AV	I	PA	NP
**Heller et al 2 cas (7)**	2000 2000	55 52	SV PV	PAV PAV	7 X 4 2,5	urethrale urethrale	NP NP	RCC RT + LE	NP 5ans/ Pas de récidive tumorale
**Mudhar et al (8)**	2001	56	PV	PPV	1	AV	I	LE	1 an / Pas de récidive tumorale
**TJALMA et Colpaert (9)**	2006	55	SV	PPV	4.5 x 4 x2.7	AV	II	PA	20 mois/ Pas de récidive tumorale
**Ditto et al (10)**	2007	53	Fortuite	PPV	1x2	NP	NP	LE + CMT	32 mois/ Pas de récidive tumorale
**DRISS et al (11)**	2007	70	Hématurie	PAV + meat uréthral	4	AV	NP	Refus du traitement	NP
**Notre cas**	2012	46	PV	PPV	5	NP	III	RCC + RIS et de la PPV + CMT	2 ans/Métastases osseuses

**SV:** Saignement vaginal. **PV:** Pertes vaginales. **PAV:** Paroi antérieure du vagin. **PPV:** Paroi postérieure du vagin. **AV:** Adénose vaginal. **NP:** Non précise. **PA:** Pelvectomie antérieure. **CMT:** Chimiothérapie. **LE:** locale Excérese. **RT:** Radiothérapie. **RIS:** Résection inter-sphinctérienne. **RCC:** Radiochimiothérapie concomittante.

Histologiquement le vagin est revêtu d'un épithélium malpighien non kératinisé et dépourvu de glandes ce qui rend mystérieux l'origine de ces ADK. L'adénose vaginale a été définie comme la présence de l′épithélium glandulaire dans le vagin à cause de la séquestration de l’épithélium glandulaire mullerian au sein de la muqueuse vaginale au cours de l′embryogenèse [12]. Il est estimé qu′environ 8% des femmes de moins de 25 ans ont une adénose vaginale [12]. Déjà en 1965 Sandberg avait suggéré que l′adénocarcinome vaginal pourrait survenir à partir des foyers d'adénose [13]. Ce tissu d'origine mullerian peut subir une métaplasie intestinale, puis une dysplasie et à la fin un adénocarcinome invasif. Plusieurs cas d'adénomes tubulo-villeux de type entérique ont été rapportés dans le vagin, la vulve et la cloison recto-vaginale [5, 14, 15].

Histologiquement, ces adénomes sont identiques à leurs homologues du côlon et sont caractérisés par des prolongements papillaires en doigt de gants avec un stroma fibro-vasculaire [8]. Un des facteurs qui causent la métaplasie est l'environnement acide du vagin [1].

Une autre explication de l′origine des adénocarcinomes entériques dans le vagin est la théorie concernant le développement à partir des restes cloacaux [5, 8, 16]. Au cours du processus de développement par voie vaginale, le septum uro-génital va diviser le cloaque en une partie antérieure (sinus urogénital primitif) et une partie postérieure (le canal anorectal), entre lesquelles le périnée est formé. Les restes cloacaux sont en fait des zones de développement des tissus anorectaux incorporés dans la paroi vaginale postérieure au cours de ce processus [8]. Le milieu vaginal acide est susceptible de causer la transformation maligne de cet épithélium intestinal. Renforçant cette théorie, il a été rapporté une description d′une muqueuse intestinale normale de type épithélium cylindrique adjacente à un normal épithélium pavimenteux dans le vagin, qui a progressé à travers la dysplasie vers l'ADK [16]. Parmi les 9 cas d'ADK primitif du vagin de type intestinal qui ont été publié, ont été rapportés 5 cas de dégénérescence d'un adénome tubulo dysplasique vers un adénocarcinome, 2 cas dont l'origine urèthral a été suggéré vue que la lésion tumorale siège en péri-urétrale, 1 cas dans l'origine cloacale a été évoqué et dans la lésion a été présenté sous forme d'un polype dégénéré (Tableau 1). Chez notre patient la lésion siégeait au niveau de la jonction tiers moyen/ tiers inférieur de la paroi postérieure du vagin. C'est une situation intermédiaire entre le vagin et rectum, ce qui laisse suggérer pour certain auteurs que la tumeur pourrait provenir de la cloison recto-vaginale et infiltre ensuite la muqueuse vaginale [17, 18]. L'ADK a été de type colloïde muqueux, sans signe de dégénérescence d'un adénome dysplasique.

Par définition d'un ADK primitif du vagin, il faut éliminer la présence d'autres sites d'ADK qui envahirent le vagin soit par contigüité (Vulve, col utérin, endomètre, urètre ou rectum) ou par une métastase à distance (rénale, sein, colon, poumon ou autres origines).Plusieurs marqueurs immuno-histochimiques sont utilisés pour différencier les tumeurs provenant de différents sites primaires. Surtout, la combinaison de CK7 et CK20 est souvent utile. Les ADK d'origines mammaires et pulmonaires sont fortement positive au CK 7 et expriment faiblement le CK20. Par contre dans les ADK d'origine colique ils sont constamment positifs au CK20 et ne sont que rarement CK7 immuno-réactive. Dans le cas présent, le carcinome intestinal ét*ait immuno-réactive au CK 20 et négatif pour le CK7. Toutefois, l′expression du profil cytokératine n′est pas utile pour distinguer une tumeur métastatique d′une tumeur primitive. Le diagnostic d′un ADK du vagin de type intestinal ne peut être retenu qu'après une vaste enquête étiologique incluant: un examen clinique approfondi, une recto- coloscopie, une TDM-TAP et éventuellement une IRM pelvienne. Chez notre patiente un bilan exhaustif a été réalisé, incluant tous les examens sus décrits, et qui n'a pas révélé d'autres localisations tumorales, ce qui nous a ainsi permis de retenir le diagnostic d'un ADK primitif du vagin de type intestinale.

Le cancer du vagin est répandu comme une tumeur de mauvais pronostic, surtout que la découverte de cette tumeur se fait généralement à un stade avancé [3, 19]. De plus, l′ADK du vagin est de plus mauvais pronostic que le carcinome épidermoïde [20]. En raison de la rareté de cet ADK type intestinal du vagin, la gestion optimale est inconnue et les résultats sont très variables. Concernant les 9 cas d'ADK vaginale de type intestinal, rapporté dans la littérature et à part un cas de récidive tumorale à 8mois après une exérèse locale suivie d'une radiothérapie [5], le traitement chirurgical semble donner de bons résultats en association avec la radio et/ou la chimiothérapie.

En résumé, nous avons rencontré un cas extrêmement rare d′ADK vaginal primaire chez une patiente jeune, traité par exérèse chirurgicale large après une RCC, suivi d'une CMT adjuvante. La pièce opératoire a été complètement stérilisée par la radio-chimiothérapie. Cependant sur un suivi de 24 mois on a été surpris par une récidive tumorale osseuse disséminée, confirmant ainsi le mauvais pronostic de ces tumeurs.

## Conclusion

Le type intestinal de l'Adénocarcinome primitif du vagin est extrêmement rare. Le diagnostic ne peut être établi que sur une étude immunohistochimique et après avoir éliminé les autres sites tumoraux de ce type histologique. Il n'y a pas de consensus concernant leur prise en charge thérapeutique. Cette observation illustre la difficulté diagnostic et le mauvais pronostic de cette tumeur maligne.
